# A Highly Sensitive Ammonia Gas Sensor Using Micrometer-Sized Core–Shell-Type Spherical Polyaniline Particles

**DOI:** 10.3390/s21227522

**Published:** 2021-11-12

**Authors:** Masanobu Matsuguchi, Tomoki Nakamae, Ryoya Fujisada, Shunsuke Shiba

**Affiliations:** Department of Materials Science and Biotechnology, Graduate School of Science and Engineering, Ehime University, Bunkyo-cho 3, Ehime, Matsuyama 790-8577, Japan; xsbry422@outlook.jp (T.N.); i844027m@mails.cc.ehime-u.ac.jp (R.F.); shiba.shunsuke.yu@ehime-u.ac.jp (S.S.)

**Keywords:** polyaniline, NH_3_ gas sensor, microspheres, core–shell structure

## Abstract

A highly sensitive NH_3_ gas sensor based on micrometer-sized polyaniline (PANI) spheres was successfully fabricated. The PANI microspheres were prepared via a facile in situ chemical oxidation polymerization in a polystyrene microsphere dispersion solution, resulting in a core–shell structure. The sensor response increased as the diameter of the microspheres increased. The PSt@PANI(4.5) sensor, which had microspheres with a 4.5 μm average diameter, showed the largest response value of 77 for 100 ppm dry NH_3_ gas at 30 °C, which was 20 times that of the PANI-deposited film-based sensor. Even considering measurement error, the calculated detection limit was 46 ppb. A possible reason for why high sensitivity was achieved is simply the use of micrometer-sized PANI spherical particles. This research succeeded in providing a new and simple technology for developing a high-sensitivity NH_3_ gas sensor that operates at room temperature.

## 1. Introduction

Ammonia (NH_3_) gas is not only a common air pollutant but is also expected to be an important energy source in the coming hydrogen society [1,2]. In line with this societal trend, environmentally friendly, low-cost methods for producing ammonia have been studied [3,4]. In application areas such as manufacturing plants and transportation processes, ammonia gas needs to be monitored for leaks because of its toxicity and adverse effects on the environment [5]. Moreover, NH_3_ gas is known as a medical biomarker for kidney disorders and *Helicobacter pylori* bacteria-induced stomach infections [5,6]. Therefore, ppb-level NH_3_ gas is of great significance for medical diagnostics.

In light of this background, there has been a demand for NH_3_ gas sensors that are not only highly sensitive, but also simple, low-cost, and non-heatable. Polyaniline (PANI) has the potential to realize such requirements [7,8] and has long been studied as a NH_3_ gas sensing material due to its easy synthesis, unique doping/dedoping processes, and good environmental stability. Moreover, PANI displays stable electrical conduction at room temperature, which is advantageous for reducing the power consumption of the sensor and for increasing portability. PANI can be synthesized easily by chemical oxidative polymerization of aniline hydrochloride [9]. However, the sensing characteristics of a sensor prepared by drop-coating an insoluble PANI dispersion solution on a sensor substrate were insufficient for practical use. This is because the formed film had a granular structure consisting of aggregated polyaniline chains, resulting in a dense film with poor gas diffusivity. The formation of thin films is expected to decrease the diffusion distance of gas molecules, resulting in a shorter response time. In general, however, reducing the film thickness also reduces the magnitude of the sensor response.

There are two main research approaches to realize the detection of NH_3_ gas at the ppb level. The first approach is a preparation of PANI nanocomposite materials. Various composites of PANI with carbon nanotubes or graphene [10,11,12,13,14], metal oxide semiconductors [15,16,17], and polymer fibers [18,19,20] have been reported to be effective for enhancing sensitivity. As an alternative approach, it has been widely reported that developing structure-controlled PANI films with large surface areas and good gas diffusivity, such as nanofibers [21,22,23,24,25], nanorods [26], and nanotubes [27], is an effective way to improve gas sensing properties.

The key idea of the present study is to use micrometer-sized PANI spherical particles for the sensing film. Films consisting of spherical particles of uniform size are expected to have high gas diffusivity because of the large amount of space inside the film. Moreover, the net volume of the space between spherical particles is expected to increase as the size of the spheres increases. Therefore, we came up with the idea of producing micrometer-sized PANI spherical particles. The benefit of this approach is that it leads to the enhancement of the interaction between PANI chains and gas molecules, resulting in the achievement of ppb-level gas detection. However, it is hard to produce spherical particles of uniform size only with PANI. Wallace et al. reported that polyaniline nanoparticles were synthesized by emulsion polymerization inside dodecylbenzenesulfonic acid (DBSA) micelles [28]. However, the nanoparticles were not perfect spheres, and they were several tens of nanometers in diameter. If particles are neither spherical nor small, the density of the formed film will increase, and improvement of gas diffusivity cannot be expected. An alternative way to reliably obtain PANI spherical particles is to coat conventional spherical polymers with PANI. Many so-called core–shell types of conducting polymers have been reported to improve processability and to coat particles [29,30,31,32,33]. Among these spherical particles, we have chosen PANI-coated PS microspheres because polystyrene microspheres can be easily prepared via a free-radical-initiated polymerization method such as dispersion polymerization. Moreover, by optimizing the polymerization conditions, the particle size can be tuned within a wide diameter range [34,35]. PANI-coated PS spheres have been subjected to electrochemical measurements [36,37], but few examples of applications to gas sensors have been reported so far. Li et al. prepared PANI nanoparticles, each composed of a PANI shell and a sulfonated PSt core, and extracted the core to make them hollow [38]. The hollow nanoparticles had a rambutan-like structure, and the hollow part was 250 nm, with a nanorod length of 100 nm. The NH_3_ gas sensing characteristics of the hollow nanoparticles at room temperature included high response values and fast response/recovery times.

In this paper, we report the results of a further improvement in sensitivity by preparing micrometer-order PANI spherical particles and investigating the effect of particle size on the NH_3_ gas sensing response.

## 2. Materials and Methods

### 2.1. Materials

The following reagents were used for the synthesis of polystyrene microspheres. Styrene as a monomer, methanol and ethanol as solvents, and polyvinylpyrrolidone as a dispersion stabilizer were obtained from Fujifilm Wako Pure Chemical. Azobisisobutyronitrile as a polymerization initiator was obtained from Sigma-Aldrich Japan. For the polymerization of polyaniline, aniline hydrochloride monomer and ammonium persulfate as an oxidant were obtained from Fujifilm Wako Pure Chemical. The styrene monomer was distilled under reduced pressure before polymerization to remove the polymerization inhibitor. Other reagents were of analytical grade or better and were used as received.

### 2.2. Preparation of Polystyrene Microspheres

A dispersion polymerization was performed to prepare micrometer-sized polystyrene spherical particles. The size of polystyrene microspheres can be controlled by the concentration of the dispersion stabilizer, the reaction solvent, the volume of the reaction vessel, and the polymerization temperature [34,35]. In this study, four sizes of microspheres were prepared. Each is hereafter referred to as Samples 1–4, respectively.

As an example of a synthesis procedure, the preparation of Sample 2 is described here. First, a 90 mL ethanol solution containing 2.5 w/v% polyvinylpyrrolidone (PVP) was placed in a three-necked 300 mL flask, and the mixture was stirred at 70 °C for 3 h under a nitrogen atmosphere. Then, 15 g of styrene (St), in which 0.33 g of azobisisobutyronitrile was dissolved, was added dropwise to the three-necked flask, and the mixture was stirred for 24 h. The obtained milky-white latex was centrifuged to remove the supernatant containing the unreacted materials. The precipitate was redispersed in ethanol and centrifuged again. These procedures were repeated three times to finally obtain purified PSt microspheres.

### 2.3. Preparation of the Core–Shell Type of Polyaniline Microspheres

Chemical oxidation polymerization of aniline was performed in a suspension of aqueous solution of PSt microspheres having different sizes, and then the surface of the PSt core was coated with polyaniline [36]. Microspheres with a structure consisting of a PSt core and a PANI shell are referred to as PSt@PANI(x) hereafter. Here, “x” represents the average diameter of PANI-coated PSt microspheres in μm. To make the thickness of the PANI shells of all microspheres uniform, the concentration of the suspension of PSt microspheres was adjusted so that the total surface area of PSt microspheres in the solution was identical.

A typical preparation procedure of PSt@PANI(2.5) using the Sample 2 microspheres was as follows. First, 100 mL of a suspension of 1.6 wt% PSt microspheres was prepared. Next, 1.11 g of aniline hydrochloride was added to the solution and cooled in an ice bath at 0 °C for 2 h. Ammonium persulfate was then added, and polymerization was performed at 0 °C for 5 h and further at room temperature for 24 h. The obtained dispersion solution of PANI-coated microspheres was centrifuged to remove the supernatant, followed by dispersion in distilled water, and then centrifuged again. This centrifugation procedure was repeated until the supernatant became colorless and transparent. The obtained microspheres coated with HCl-doped PANI were dispersed in 25% aqueous ammonia and stirred for 24 h for the de-doping of HCl. Then, the microspheres were dispersed in 0.025 M of sulfuric acid, stirred for 24 h for doping again, and finally microspheres coated with H_2_SO_4_-doped PANI were obtained.

### 2.4. Preparation of Sensing Films

The dispersion aqueous solution of H_2_SO_4_-doped PANI microspheres was drop-coated onto an alumina substrate having a pair of interdigitated gold electrodes with an electrode spacing of 200 μm (Figure S1). The film was heat-treated at 150 °C for 20 min in air.

### 2.5. Measurement of NH_3_ Gas Sensing Properties

All sensors were set in a home-made thermostated measuring cell. The experimental setup is shown in Figure S2. Measurement was performed at 30 °C in flows of different concentrations of NH_3_ gas diluted with N_2_. The variation in the electrical resistance was measured with an electrometer (8252, ADC). Baseline measurements (*R*_0_) were performed in nitrogen, followed by measurement of the resistance of the sensor in NH_3_ (*R*_g_) in various concentrations. The magnitude of the sensor response was defined as

*S* = *R*_g_/*R*_0_. (1)

Response (*T*_res_) and recovery time (*T*_rec_) were each defined as the time required to reach 90% of the total resistance change.

### 2.6. Characterization

The morphologies of the PSt microspheres before and after PANI coating were observed using a field emission scanning electron microscope (FE-SEM) (S-5500, Hitachi High-Technologies). The X-ray diffraction (XRD) analysis was carried out on an X’pert Pro (PANalytical) with Cu Ka radiation.

## 3. Results and Discussion

### 3.1. Characterization of the Core–Shell Type of Polyaniline Microspheres

**Figure 1:**
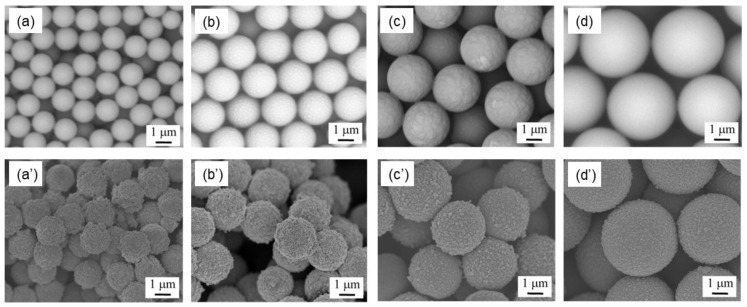
SEM images of PSt microspheres before and after PANI coating: (**a**,**a’**) Sample 1; (**b**,**b’**) Sample 2; (**c**,**c’**) Sample 3; and (**d**,**d’**) Sample 4.

Figure 1a–d shows SEM images of the PSt of Samples 1 to 4 before PANI coating. All PSt samples prepared were proved to be spherical. Their estimated diameters are summarized in Table 1, and all spheres were confirmed to be in the order of micrometers. The table also shows the coefficients of variation (CV). Since the CV values were small in all microspheres, it was confirmed that PSt microspheres with high monodispersity were obtained. SEM images of PSt microspheres after PANI coating are shown in Figure 1a’–d’. PANI homogeneously covered the surfaces of all the PSt microspheres. Coating the PSt microspheres slightly increased their diameters, and the thickness of the polyaniline shell was estimated from the difference in the microspheres’ radii from before to after the PANI coating. As shown in Table 1, in all microspheres, the polyaniline shell layer was found to be about 200–250 nm.

**Figure 2:**
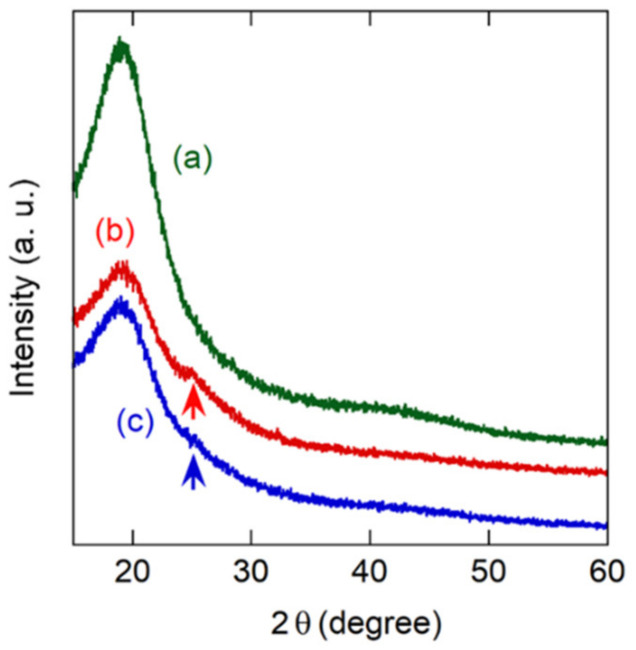
XRD patterns of (**a**) PSt(4.1) microspheres, (**b**) PSt@PANI(2.5) microspheres, and (**c**) PSt@PANI(4.5) microspheres.

It is well known that the crystallinity of PANI has a significant effect on conductivity and gas sensor characteristics [39,40]. The crystal structure of PANI coated on a microsphere surface was confirmed by XRD measurement. The X-ray diffraction patterns obtained by PANI-coated microspheres with different particle sizes are shown in Figure 2. It was shown that each PANI microsphere had a broad diffraction peak of approximately equal intensity centered at 2*θ* = 20°. However, the peak is also seen in PSt microspheres and is reported as a typical XRD feature of amorphous PSt [41]. Crystallized PANI is known to have a sharp peak at 2*θ* = 25.6°, which is derived from the periodicity perpendicular to the PANI polymer chains [42,43]. The corresponding peak was seen in both PANI microspheres, indicating the semi-crystalline phase. However, the peak was very weak and broad, suggesting low crystallinity of PANI on the surface of each microsphere. This result is natural because the coating method and conditions were the same; only the core size was different.

### 3.2. Sensing Properties toward NH_3_ Gas

NH_3_ gas adsorption on the PANI-coated microspheres led to a large increase in electrical resistance. Then, after the sensor was exposed to dry N_2_, the resistance reverted toward the initial value. This reversible response of PANI to NH_3_ gas can be explained by a well-known sensing mechanism: the process of protonation−deprotonation (Figure S3) [40].

**Figure 3:**
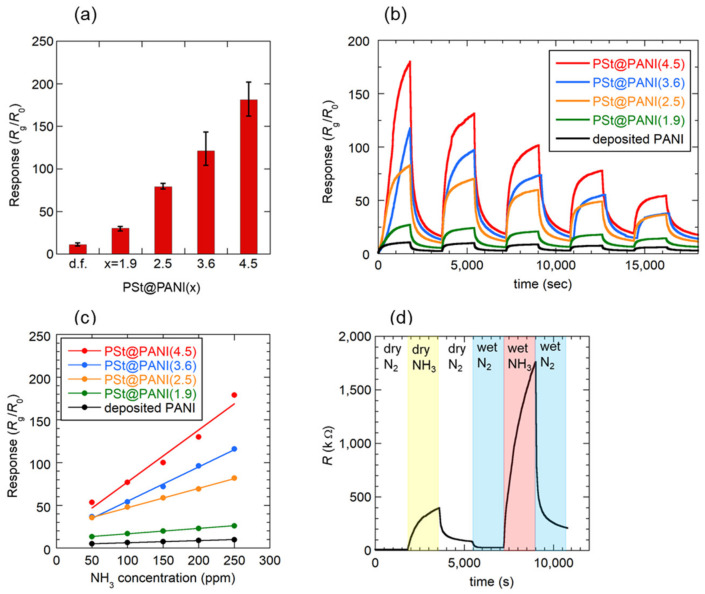
NH_3_ gas sensing characteristics obtained for the PSt@PANI(x) sensor: (**a**) magnitude of the sensor response toward 250 ppm dry NH_3_, (**b**) transient response curves when the dry NH_3_ gas concentration is reduced by 50 ppm from 250 ppm to 50 ppm, (**c**) calibration curves between 50–250 ppm dry NH_3_ gas, and (**d**) effect of the humidity (50% RH) on the sensor response of the PSt@PANI(1.9) sensor. All measurements were performed at 30 °C.

A comparison of the magnitudes of the responses obtained for the PSt@PANI(x) sensors is shown in Figure 3a. For this purpose, sensors with similar sensor resistances of about 100 kΩ were selected. The polyaniline-deposited film (d.f.) formed by the conventional drop-cast method is also shown. As expected, the sensor using microspheres showed a greater sensor response than the sensor using the conventional drop-casting film. Among the microsphere-based sensors, PSt@PANI(4.5) showed the greatest sensor response, which was 20 times higher than that of the sensor based on deposited film. As shown in Figure 2, it is unlikely that the difference in the crystallinity of PANI had a significant effect on the sensor response with different microsphere sizes. Consequently, as will be explained later, this enhancement of the sensor response can be expected to be closely related to the size of the microspheres. In this study, PANI microspheres with a diameter larger than 4.5 mm were not examined because of the difficulty of preparing larger PSt microspheres.

Figure 3b exhibits the transient response curves of each sensor when the NH_3_ gas concentration was reduced by 50 ppm from 250 to 50 ppm. All sensors responded reversibly to ammonia gas, showing faster response and reversibility, especially at lower concentrations. However, the estimated response and recovery times at 50 ppm were *T*_res_ = 700 s and *T*_rec_ = 1050 s, respectively, which were longer than those of the PANI-based sensors reported so far. The reason for this apparently slow response is that in our measuring setup, it takes a long time to replace the gas in the measuring cell, so that it is not possible to distinguish between the actual response time and the gas filling time. Therefore, the response/recovery times estimated in this study may be longer than the actual time.

The ammonia gas concentration dependence of the sensor response is shown in Figure 3c. Although the response of each sensor changed linearly within the measured NH_3_ gas concentration range, the sensitivity estimated from the slope of the straight line was the highest for the film consisting of PSt@PANI(4.5), which was 25 times that of the conventionally deposited film sensor. The response value for PSt@PANI(4.5) was *S* = 77 toward 100 ppm NH_3_ at room temperature. Due to the very large sensitivity, the limit of detection was estimated using Equation (2) considering the standard deviation *σ* of the base line, that the slope of the calibration curve was 46 ppb, and expecting that ammonia gas can be detected at the sub-ppm level.

LOD = 3.3*σ*/slope (2)

It is well known that humidity affects the electrical conductivity of PANI, and its application to a humidity sensor has been studied [44]. Therefore, atmospheric humidity also affects the characteristics of a PANI-based NH_3_ gas sensor [15,40,45]. Figure 3d shows the results of an investigation into the effect of relative humidity (%RH) on the response of the PSt@PANI(1.9) sensor. After the sensor response to NH_3_ gas was measured for one cycle in a dry atmosphere, switching from dry N_2_ to wet N_2_ (50% RH) reduced the electrical resistance. The cause of this resistance reduction has been explained as a “proton effect”, that is, doping with protons generated by the dissociation of water molecules [11,44]. Subsequent exposure to NH_3_ gas humidified to 50% RH significantly increased the resistance. The resulting response value was *S* = 62, which was 1.7 times larger than that of *S* = 37 in a dry atmosphere. Many groups have reported a complex mechanism underlying humidity’s effect on the NH_3_ gas sensing of PANI. The increase in the response value can be explained by the “swelling effect” [44,46] and/or the capture of protons from the acidified PANI by OH^−^ generated through the following reaction [38]:

NH_3_ + H_2_O ⇄ NH_4_^+^ + OH^−^ (3)

On the other hand, there are reports that the response value decreased again at higher humidity (60% RH or higher), and this phenomenon was explained by the reduction of adsorption sites of NH_3_ by the competitive adsorption of NH_3_ and excess H_2_O [11,13,46,47].

In this study, the selectivity to other gases was not investigated. However, many papers have reported that PANI-based gas sensors had excellent selectivity toward NH_3_ gas and showed low responses to other volatile organic vapors and some common toxic gases [18,38,40]. Our sensors can be expected to possess this property of high selectivity, as the structure and the dopant of the PANI we used are not special.

To estimate the performance of the present PSt@PANI(4.5) sensor, the magnitudes of response of previously reported high-sensitivity NH_3_ gas sensors using PANI are summarized in Table 2. In this table, some response values were recalculated to match our definition *S* = *R*_g_/*R*_0_. It should also be noted that many of the responses are values obtained in a humidified atmosphere. Therefore, there is a problem in that the detection performance of the sensors for NH_3_ gas alone cannot be compared. In general, such comparisons should be made on the assumption that the sensor responses obtained in a humidified atmosphere can be considered larger than those obtained in a dry atmosphere. Wojkiewicz et al. reported two systems of core–shell-type PANI sensor: one with a poly (vinylidene fluoride) (PVDF) core and the other with a poly (butyl acrylate) (PBuA) core [48]. A relatively porous film was formed with an average particle size of around 50–100 nm for PVDF@PANI, but adhesions between particles were observed. Regarding the PBuA@PANI, the nanoparticles deformed into two-axial ellipsoids and a film consisting of densely packed particles formed. Thus, considering that these sensor responses are measured under a humidified condition, these sensors did not provide a large sensor response compared to our PSt@PANI(4.5) sensor. Li et al. prepared hollow polyaniline nanospheres by coating sulfonated PSt cores with PANI and then extracting the PSt cores [38]. Interestingly, this resulted in a rambutan shape with a hollow sphere diameter of 250 nm, which was one-tenth that of our microspheres, and a nanorod array length of 100 nm. The authors stated that this characteristic shape had the effect of increasing the surface area and increasing the number of adsorption sites. Unfortunately, however, this rambutan-like nanosphere alone could not sufficiently increase the sensor response. High sensitivity was obtained by making it a hybrid with graphene oxide (GO). Since pure PANI had poor sensor performance, many reports utilize the synergistic effect of p-type PANI and nano-structured n-type metal oxide semiconductors [45,49,50,51]. PANI-coated Au-loaded mesoporous In_2_O_3_ nanospheres showed a high response value (*S* = 46 at 100 ppm in 50% RH) [45]. The In_2_O_3_@PANI nanospheres ranged in diameter from 130 nm to 160 nm, more than an order of magnitude smaller than our microspheres. A sensor that was particularly sensitive to low-concentration NH_3_ (*S* = 9.8 at 3 ppm) gas was obtained by growing PANI-CSA/TiO_2_ on the inner wall of a glass tube [50]. More recently, the results of actually measuring the sensor response in an extremely low-concentration atmosphere of 1 ppm or less have been reported. PANI-SrGe_4_O_9_ nanocomposites had a unique hierarchical architecture with abundant mesopores [51]. The sensor showed an ultralow detection limit of 0.25 ppb toward NH_3_ in 60% RH, although the sensor response was *S* = 3.1 for 10 ppm NH_3_, which was small compared to that of the present PSt@PANI(4.5) sensor. Since there are some unclear points in the calculation of the detection limit, such as the inclusion of measurement error, a comparison of the reported LOD values should be done carefully. In addition, electrochemically synthesized PANI on commercial screen-printed three-electrode systems succeeded in detecting 32 ppb in 70% RH, and the calculated LOD according to the formula from IUPAC recommendations was 23 ppb [52]. Compared to the sensors reported above, our PANI microsphere sensor yielded greater sensitivity (*S*/Concentration) without utilizing the synergistic effect with metal oxide semiconductors or carbon nanomaterials and without using a special coating method or equipment.

### 3.3. Mechanism of Response Increase

We next discuss the reason why sensitivity was greatly improved by using the PANI microspheres. It is unlikely that the difference in the crystallinity of PANI due to the difference in the diameter of the microspheres affected the response value, as explained in Section 3.1.

**Figure 4:**
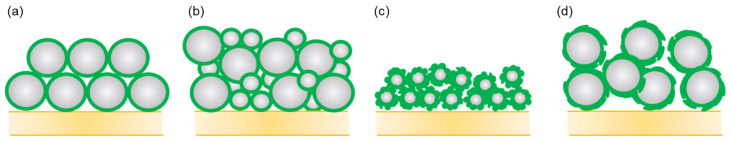
Cross-sectional illustration of core–shell-type PANI-sphere-deposited film: (**a**) close-packed structure consisting of uniform sized spheres, (**b**) structure consisting of irregularly sized spheres, (**c**) actual structure consisting of uniform nanometer-sized spheres, and (**d**) actual structure consisting of uniform micrometer-sized spheres.

**Figure 5:**
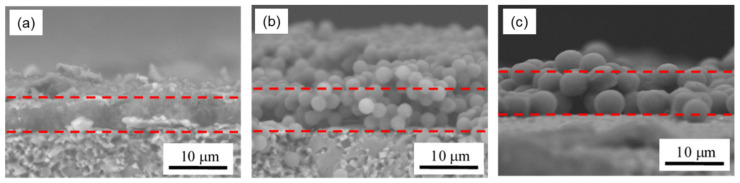
SEM images of cross sections of sensors: (**a**) deposited film, (**b**) PSt@PANI(2.5) film, and (**c**) PSt@PANI(4.5) film.

Another possible reason for the improvement is that the size of the spherical particles affected the response value. It should be noted here that the electrodes were located below the sensing film. It can be theoretically calculated that the spheres occupy 74% of the volume when the spheres are closely packed, as illustrated in Figure 4a [53]. Therefore, as a general theory, film made of spherical particles has a large amount of space inside the film that is effective for gas diffusion to reaction sites near electrodes and is thus promising as a gas sensing film. However, when the sizes of spheres are irregular, as shown in Figure 4b, the small spheres fill the spaces between the large spheres, so that the packing efficiency becomes high [53]. In this regard, it can be said that producing spherical PANI particles having a uniform size was effective in increasing the sensor response. It is also important to explain why a larger response was obtained by simply increasing the size of the spheres. When PANI coats on the surface of nanometer-sized spheres, it is difficult to form a uniformly thin PANI shell layer on such tiny cores. As a result, PANI-coated particles have irregular shapes, and adhesions between nanospheres are likely to occur [48]. In fact, as can be seen in Figure 1, even in the case of microspheres, the smaller the particle size, the coarser the surface coating. Particle size miniaturization is generally effective at increasing total surface area but produces a trade-off effect in this case of increasing the packing efficiency (Figure 4c) and suppressing the diffusion of gas molecules to the reaction sites. Of course, when the microspheres are actually coated on the substrate, the arrangement of the spheres becomes more random, and the packing density is lower than in the case of dense packing (Figure 4d). To confirm the density of the PSt(x)@PANI films, cross sections of the sensors were observed with SEM and are shown in Figure 5. The drop-casting film formed a very dense structure in which irregularly shaped PANI nanoparticles of 100–200 nm were aggregated. On the other hand, the structure of the film consisting of PANI microspheres contained a lot of space inside. Moreover, microspheres with an average diameter of 4.5 μm had more internal space due to the larger particle size, as expected. As a result, most of the PANI thin film present in the shell layer of the spheres was exposed to NH_3_ gas. Thus, it is considered that a large sensor response was obtained.

## 4. Conclusions

NH_3_ gas sensors based on core–shell-type PANI microspheres were prepared. The present preparation method of PANI microspheres was very easy and did not require any special equipment. It was confirmed that the sensor response increased as the diameter of the microspheres increased. The present (PSt@PANI(4.5)) microsphere-based sensor showed higher sensitivity than the previously reported sensors based on typical structure-controlled PANI and/or PANI composites toward NH_3_ gas at room temperature. This success in increasing the sensor response may have a very simple explanation: by producing a sensing film made of PANI uniformly coating micrometer-sized spheres, many spaces formed inside the film, resulting in the enhancement of the reaction between NH_3_ molecules and PANI. It is an important result of this study that our sensor achieved a greater sensor response with pure PANI by simply increasing the size of the core spheres. PSt@PANI microsphere sensors have advantages such as ease of preparation, low cost, and mass producibility. However, the response of this sensor was affected by humidity, as were the other PANI-based sensors, so it is necessary to take some measures against that effect. In the future, we intend to reduce the response time and increase the sensitivity by further optimizing the size of the spherical particles and by further reducing the thickness of the polyaniline shell.

## Figures and Tables

**Table 1:** Average diameter (AD) and coefficient of variation (CV) of PSt- and PANI-coated PSt microspheres, and estimated thickness of PANI shell layer.

	PSt		PANI-PSt		Thickness of PANI Shell Layer (nm)
Sample No.	AD (μm)	CV (%)	AD (μm)	CV (%)	
1	1.4	3.4	1.9	6.3	250
2	2.0	1.4	2.5	3.5	250
3	3.1	7.2	3.6	10.7	250
4	4.1	6.9	4.5	9.0	200

**Table 2:** Comparison of PANI-based NH_3_ gas sensing performances of the present sensor with other previously reported sensors.

Material	Features	Temp. (°C)	Humidity (% RH)	Conc. (ppm)	*S* ^(1)^	*S*/Conc. (ppm^−1^)	LOD (ppb)	Ref.
PSt@PANI(4.5)	Core–shell	30	dry	100	77	0.77	46	This work
PSt@PANI(1.9)	Core–shell	30	dry	250	37	0.15		This work
PSt@PANI(1.9)	Core–shell	30	50	250	62	0.25		This work
PVDF@PANI	Core–shell	25	50	10	2 ^(2)^	0.20	100	[48]
In_2_O_3_@PANI	Core–shell	20	50	100	46	0.46	500	[45]
GO/PANI	Hollow nanosphere	20	25	100	32	0.32	50	[38]
WO_3_@PANI	Nanoplate	25	40	100	34	0.34		[49]
PANI/TiO_2_	Tube	R.T.	dry	100	17 ^(2)^	0.17		[50]
PANI/Ti_3_C_2_T_x_	Flexible	20	dry	50	4.0 ^(2)^	0.08	25	[47]
PANI/cellulose	Nanofiber	R.T.	45	100	6.1	0.06	200	[40]
PANI/SrGe_4_O_9_	Nanocomposite	25	60	10	3.1 ^(2)^	0.31	0.25	[51]
PANI on SPEs	Electrochemically deposited	25	70	0.5	1.0	2.0	23	[52]

^(1)^ *S* = *R*_g_/*R*_0_. ^(2)^ Value recalculated according to definition.

## Data Availability

Not applicable.

## Supplementary Materials

The following are available online at https://www.mdpi.com/article/10.3390/s21227522/s1, Figure S1: Illustration of an alumina substrate having a pair of interdigitated gold electrodes, Figure S2: Experimental setup used for NH_3_ gas sensing measurement, Figure S3: Sensing mechanism of PANI-based NH_3_ gas sensors.
