# Supplementary material for: A Highly Sensitive Ammonia Gas Sensor Using Micrometer-Sized Core–Shell-Type Spherical Polyaniline Particles
Source: Sensors (Basel). 2021 Nov 12;21(22):7522. doi: 10.3390/s21227522 (PMC8619626; doi:10.3390/s21227522)
Supplement: Supplementary file 1 [file sensors-21-07522-s001.zip › sensors-1455911-supplementary.pdf]

Supplementary Materials

Highly Sensitive Ammonia Gas Sensor Using Micrometer-Sized Core-Shell Type Spherical Polyaniline Particles

Masanobu Matsuguchi*, Tomoki Nakamae, Ryoya Fujisada, and Shunsuke Shiba

Department of Materials Science and Biotechnology, Graduate School of Science and Engineering, Ehime University, Bunkyo-cho 3, Matsuyama, Ehime 790-8577, Japan; matsuguchi.masanobu.mm@ehime-u.ac.jp

* Correspondence: matsuguchi.masanobu.mm@ehime-u.ac.jp; Tel.: +81-89-927-9933

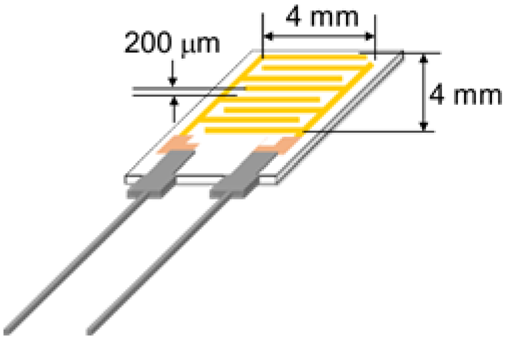

Figure S1. Illustration of an alumina substrate having a pair of interdigitated gold electrodes.

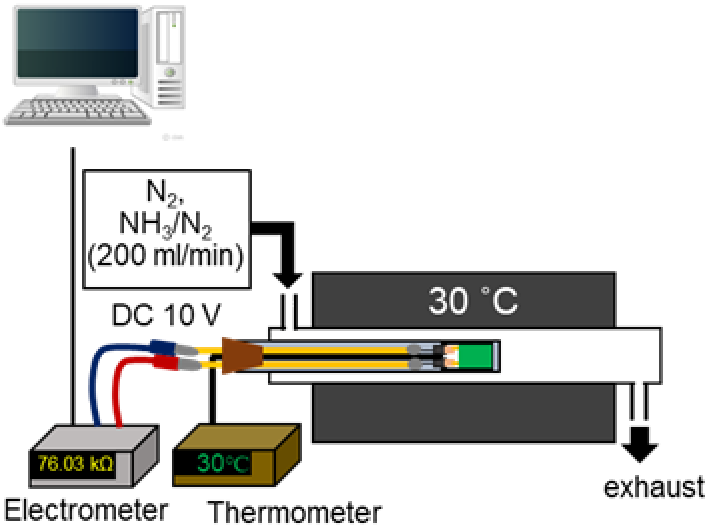

Figure S2. Experimental setup used for NH_3 gas sensing measurement.

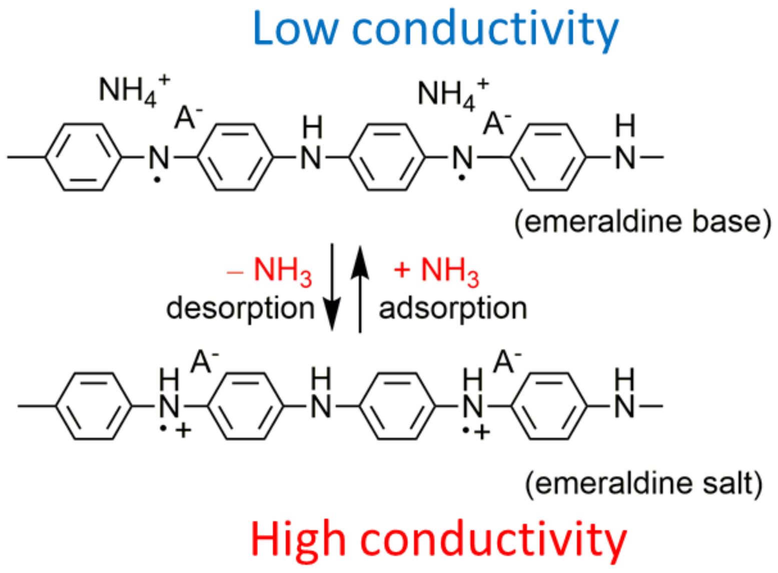

Figure S3. Sensing. mechanism of PANI-based NH_3 gas sensors.
